# Insulin in aluminum phosphide poisoning: A systematic review of the current literature

**DOI:** 10.1097/MD.0000000000040066

**Published:** 2024-10-18

**Authors:** Ravi Shukla, Kiran Lamichhane, Dhritee Pandey, Chandan Kumar Gupta, Sashank Shukla

**Affiliations:** a Nepal Medical College and Teaching Hospital, Kathmandu, Nepal; b Nepal Army Institute of Health Sciences, Kathmandu, Nepal.

**Keywords:** aluminum phosphide, insulin, pesticides, phosphines, poisoning, review

## Abstract

**Background:**

Aluminum phosphide (AlP) is a commonly used fumigant in agriculture and grain preservation because of its high potency and low cost. Due to the absence of a specific antidote and promising treatment modality, poisoning with this substance is deadly. Amid multiple studies in different parts of the world, each exploring options like lavage, inotropes, antioxidants, etc, we conducted a systematic review to find the possible role of exogenous insulin in treating symptomatic cases of AlP poisoning.

**Methods:**

Experimental studies released before February 15, 2024, that reported the use of exogenous insulin were systematically reviewed following the Preferred Reporting Items for Systematic Review and Meta-Analysis statement. The search was done on PubMed Central, Cochrane Library, and Google Scholar.

**Results:**

After applying the inclusion and exclusion criteria, we finalized a few scientific papers for the review. Studying data from 4 scientific papers (3 quasi-experimental studies and 1 randomized controlled trial), we could postulate the significant improvement in survivability after the inclusion of exogenous insulin in the treatment of poisoned cases of AlP. Studies showed divergent results for the blood pressure, blood gases, and need for mechanical ventilation. Hypoglycemia, hyperglycemia, and hypokalemia were the reported adverse effects of this therapy.

**Conclusion:**

Our review found that the use of exogenous insulin in AlP poisoning reduced mortality rates, which was consistent across all studies. With available knowledge, its inclusion as a part of therapy might be beneficial in AlP poisoning, but to put it forward confidently, we still need high-quality randomized control trials. It is indeed a subject of interest for future research.

## 1. Introduction

Aluminum phosphide (Alphos; chemical name: AlP) is a commonly used grain preservative and a lethal pesticide. The US Environmental Protection Agency has categorized AlP under Toxicity category I, the highest (most toxic) of 4 categories.^[1]^ It is common among agricultural pesticides for accidental, suicidal, and homicidal poisoning.^[2]^ Alphos is mostly marketed in the form of tablets. Poisoning can be due to intentional or accidental ingestion of tablets or inhalation of liberated toxic gas while handling. Inhalation poisoning to the health care providers while managing victims of Alphos poisoning has also been reported.^[3,4]^ The marketed AlP tablets contain AlP and aluminum carbonate.^[5]^ Toxic phosphine gas is liberated when AlP comes in contact with moisture or hydrochloric acid in the stomach. The liberated phosphine gas gets absorbed through the mucosa and produces systemic effects.^[2]^ Nausea, vomiting, fatigue, headache, difficulty breathing, and decreased level of consciousness are common clinical features at the time of presentation. Cases later complicate with congestive heart failure, cardiac arrhythmias, pleural effusion, pulmonary edema, acute respiratory distress syndrome, acute renal failure, and acute hepatic failure.^[1,6]^ Ingestion of as little as 0.5 mg of AlP is lethal. The case fatality rate has been reported to be approximately between 30% and 100%.^[7]^ The high case fatality rate of Alphos poisoning is partly attributable to its use in low-income countries and rural areas where access to critical care facilities is limited.

The pathophysiological effect of the phosphine gas is injury at the cellular level, at least by the noncompetitive blockade of cytochrome c oxidase leading to the inhibition of oxidative phosphorylation and cellular respiration and the resultant overproduction of peroxide radicals. The most deleterious effect has been seen on the myocardium. Apart from the combined effect of mitochondrial damage and oxidative injuries, the myocardium also suffers from cell membrane dysfunction and damage to proteins and cell membrane potential.^[8]^ The energy metabolism of stressed myocardium is known to shift from b-oxidation to glycolysis.^[9]^ AlP poisoning further deteriorates this scenario by decreasing the cellular uptake and metabolism of glucose. For this, some authors use the term “state of metabolic starvation.”^[10,11]^ Additionally, the significant imbalances in the body’s acid–base and electrolyte levels in AlP poisoning, such as metabolic acidosis, lactic acidosis, hypokalemia, and hyper/hypomagnesemia, which are known to independently induce myocardial dysfunction, can further exacerbate the dysfunction in already ailing myocardium.^[12]^ The most common cause of mortality in AlP poisoning is severe hypotension refractory to fluids and vasoactive agents.^[13]^ Myocardium is not the only sufferer of AlP poisoning, as phosphine is a systemic toxin and affects almost every system of the body. Death of the victims in the first few hours to days of ingestion is mostly due to cardiac compromise, so the latter effects on the other systems have been inadequately described. Nobody has confidently proposed a management modality or a potential antidote for acute poisoning with this substance, and currently, the only treatment available is supportive care. So, this has attracted numerous studies to find an effective therapy for management.

Therapeutic use of insulin in emergencies like diabetic ketoacidosis, hyperglycemic hyperosmolar syndrome, beta-blocker overdose, calcium channel blocker overdose, hyperkalemia, and hypertriglyceridemia-induced pancreatitis is almost a routine entity. Insulin as a main regulator of metabolism in cardiomyocytes, its positive inotropic activity, and cardioprotective role in cases of myocardial injuries have been studied repeatedly in the past.^[14,15]^ A possible benefit of insulin in alphos poisoning was first advocated by Hossein et al in 2008.^[16]^ Since then, a few interventional studies with insulin-based modality of treatment have been performed. Analyzing the available knowledge, theoretically, exogenous insulin use has a role in the improvement of alphos poisoning-induced circulatory compromise. Thus, we conducted this systematic review with an objective “to ascertain the outcomes of using exogenous insulin in treatment of symptomatic cases of AlP poisoning.”

## 2. Methods

### 2.1. Study design

This systematic review is reported according to the Preferred Reporting Items for Systematic Reviews and Meta-Analyses 2020 statements.^[17]^ Ethical approval was waived because the paper is a systematic review.

### 2.2. Study inclusion and exclusion criteria

All available and accessible original studies available from PubMed Central, Cochrane Library, and Google Scholar, since their inception to February 15, 2024, that discussed the use of insulin in AlP poisoning were sought. The studies that satisfied all points in the inclusion criteria and did not meet any of the points in the exclusion criteria were included in the review.

#### 2.2.1. Inclusion criteria

Large- or small-scale interventional studies on acute symptomatic AlP poisoning, regardless of age group or study site, that investigated the use of exogenous insulin either as monotherapy or as part of combination therapy. The studies should have examined at least one of the following endpoints: mortality, effects on hemodynamic parameters, or utilization of healthcare resources (e.g., mechanical ventilation, length of hospital stay).

#### 2.2.2. Exclusion criteria

(1) Studies with insufficient or unclear information.
(2) In vitro or nonhuman animal studies.
(3) Case reports, case series with fewer than 5 cases, conference abstracts, reviews, meta-analyses, letters to the editor, book chapters, theses, commentaries, newspaper articles, and other gray literature.

### 2.3. Search strategy

Standard electronic databases such as PubMed Central, Cochrane Library, and Google Scholar were searched for scientific articles. The search was carried out for published studies prior to February 15, 2024. The search used combinations of the terms “Aluminum phosphide” and “Insulin” as medical subject headings, keywords, and word variations. The adapted search strategy is available in Appendix 1, Supplemental Digital Content, http://links.lww.com/MD/N720. Apart from automated searches in databases, manual searches for scientific papers were also performed. A total of 1998 articles were found.

### 2.4. Selection process

**Figure 1.**
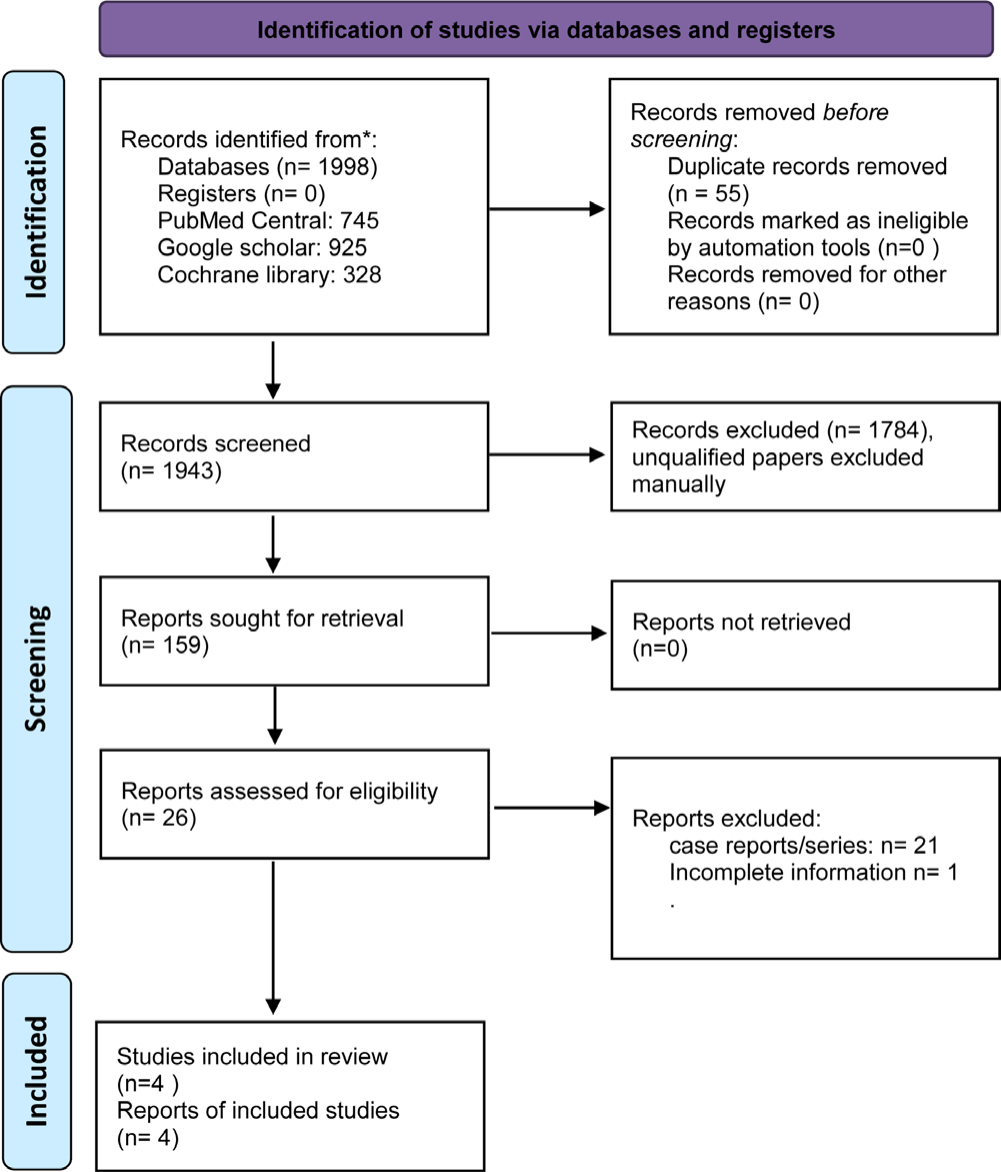
PRISMA 2020 flow diagram depicting the selection process for this study. PRISMA = Preferred Reporting Items for Systematic Reviews and Meta-Analyses.

The Preferred Reporting Items for Systematic Reviews and Meta-Analyses 2020 flow diagram of study selection appears in Figure 1. Two reviewers (RS and KL) independently screened the titles and abstracts of these papers for exclusion and inclusion. The abstracts seemingly fit for inclusion or with ambiguous information were reviewed in full-text form. The articles that were not readily accessible were accessed in Hinari through our institutional account. An overall evaluation for potential overlap of the population was conducted based on authorship, study site, and recruitment period. Before excluding papers, titles and abstracts in non-English languages were translated into English using Google Translate and sought for eligibility. Disagreements during the selection process were resolved through discussion with a third reviewer (DP). In the end, 3 prospective studies and 1 randomized controlled trial (RCT) were finalized for the systematic review in our paper.

### 2.5. Quality assessment

Two of our authors (RS and KL) independently performed the quality assessment. The risk of bias in the RCT was evaluated by RoB 2: a revised tool for assessing the risk of bias in randomized trials.^[18]^ The Rob1 tool was used for quality appraisal of quasi-randomized interventional studies.^[19]^ The NHLBI Quality Assessment Tool for Before-After (pre-post) Studies With No Control Group-tool was used for quality appraisal of the interventional study without a control group.^[20]^ The individual results were reviewed and compiled by a third author (DP).

### 2.6. Outcome definition

To evaluate the overall outcome effect of the use of high-dose exogenous insulin in victims of AlP poisoning. To mention:

*Primary outcomes*: effects on survivability.

Secondary outcomes: effects on blood pressure (BP) (systolic, diastolic, and mean arterial pressures [MAP]), blood pH, blood gases, need for mechanical ventilation, and possible side effects of insulin therapy.

## 3. Results

### 3.1. Literature search and data extraction

A total of 1998 articles were identified following a thorough database search. After the exclusion of duplicates and those not meeting inclusion criteria, 4 studies were reviewed for data collection. The characteristics of each included study are summarized in Table 1.

**Table 1:** Key characteristics of studies included in this systematic review.

Author (year of publication)	Study design	Study period	Age of participants mean ± SD/ median [IQR]	Study size	Duration of ingestion to hospital (median)	Intervention group			Control		Endpoint assessment
						Dose of AlP (in average)	Intervention therapy	Dose of insulin	Dose of AlP (in average)	Therapy	
Hossein et al (2016)	Prospective interventional study	2006–2012	26.2 ± 8.5 years	88	3 hours	4.5 [3,6] g	Gastric lavage, + supportive therapy + insulin	Initial bolus: 1 IU/kg Maintenance: 0.2–0.5 IU/kg/h.	3 [3,6] g	Gastric lavage, + supportive therapy.	Primary: mortality and their length of hospital stay.
Pannu et al (2020)	Prospective interventional study	July 2016–December 2017	31.6 ± 13.2	60	4 hours	Not available	Gastric lavage, + supportive therapy + insulin	Initial bolus: 0.1 to 0.2 IU/kg Maintenance 0.2 to 0.5 IU/kg/h.	Not available	Gastric lavage, + supportive therapy.	Primary: mortality, secondary: hospital stay, mechanical ventilation, BP, and metabolic acidosis.
B Adel et al (2023)	RCT	August 2020–December 2021	20 (17–34.25)	108	2 hours	3 (3-3) g	Gastric lavage, + supportive therapy + insulin	Initial bolus: 1 IU/kg Maintenance: 0.2 to 0.5 IU/kg/h.	3 (3-3) g	Gastric lavage, + supportive therapy. + placebo (normal saline)	Primary: mortality, secondary: hospital stay, mechanical ventilation, BP, and metabolic acidosis.
Nasrin et al (2023)	Prospective interventional study	February 2022–February 2023	32.99 ± 11.45	101	4 hours	1.77 ± 1.3 g	Gastric lavage + supportive therapy + insulin	Initial bolus: 1 IU/kg Followed by bolus doses up to 10 IU/kg	Not applicable		pH, blood gasses, hemodynamics parameters, urine output

RCT = randomized controlled trial.

### 3.2. Data extraction and analysis

Two of our authors, who were involved in data selection, performed data extraction and plotted it on a spreadsheet. The data extraction spreadsheet was created using Google LLC. The information about demographics of the studied population, applied treatment modalities, BP, durations of hospital stay, blood gases, and mechanical ventilation.

### 3.3. Study designs

Among 4 included studies, 3 were prospective interventional studies, and one was RCT. Hossein et al performed a non-blinded, conventional randomization, prospective interventional study.^[21]^ Pannu et al performed a prospective open-label pilot study.^[11]^ The study performed by Adel and his colleagues was a single-blinded randomized controlled trial.^[22]^ Whereas Nasrin et al performed a longitudinal interventional study without a control group to determine the effectiveness of different doses of insulin in AlP poisoning.^[23]^ All these studies were single-center studies, 2 belonging to Iran,^[12,21]^ one from India,^[11]^ and another from Egypt.^[22]^ The review of individual studies has been mentioned in the successive subtopics.

### 3.4. Treatment groups

Hossein et al, Pannu et al, and Adel et al divided the symptomatic cases of AlP poisoning into 2 equal groups. Both groups received similar supportive care. Intervention groups additionally received exogenous regular human insulin; the authors then compared it with control groups for various aspects of the toxidrome.^[11,21,22]^ Nasrin et al went a step further to determine the dose-dependent effect of insulin on physiology in symptomatic AlP poisoning cases.^[12]^

### 3.5. Drug and doses

Hossein et al, Pannu et al, and Adel et al had administered regular human insulin empirically as a part of treatment in the intervention group. In all of these studies, insulin was administered as an initial bolus dose followed by infusion. Pannu et al had used an initial bolus dose of 0.1 to 0.2 International unit (IU)/kg; for Hossein et al and Adel et al, the bolus dose was 1 IU/kg. The insulin infusion rate in all these studies was 0.2 to 0.5 IU/kg/h.^[11,21,22]^ Furthermore, Hossein et al mentioned increasing the insulin infusion dose up to 3 IU/kg/h to raise systolic blood pressure (SBP) above 90 mm Hg before initiating inotropic support. They also used insulin infusion along with bicarbonate (undefined doses) to correct metabolic acidosis. Nasrin et al used insulin whenever SBP fell below 90 mm Hg in patients receiving supportive care for AlP poisoning. They administered initial loading insulin at a dose of 1 IU/kg; if the SBP did not increase, the insulin was increased up to 10 IU/kg until the SBP reached more than 90 mm Hg.^[12]^ All the authors mentioned monitoring and euglycemic control of blood sugar levels by infusion of intravenous dextrose and maintenance of a physiological level of serum potassium. About discontinuing insulin-based therapy, Hossein et al mentioned gradual tapering of insulin infusion, reducing to 1/2 to 2/3 of the previous dose after correction of hypotension and acidosis, which were assessed 8 hourly.^[21]^ Pannu et al and Adel et al however did not mention information about discontinuation. The mean dose of insulin used in the intervention group was 379.3 (±164.5) IU for Pannu et al.^[11]^ For Hossein et al, the median dose was 525 IU with a range of 40 to 11,658 IU, and for Nasrin et al the dose of insulin ranged from 30 to 600 IU.^[12,21]^

The supportive modality of treatment in these studies included mechanical ventilation, inotropes, antioxidants, antiarrhythmics, and bicarbonate supplementation; the information about the indication, dosage, and duration of these therapies was poorly described in the studies.

### 3.6. Outcome measures

Before analyzing, we compared the obvious factors influencing the outcome of any poisoning, such as the amount of ingestion and the time interval between substance exposure and hospitalization. All the authors claimed statistically “non-significant” differences between the 2 study groups for the age of participants, amount of substance ingestion, and duration from ingestion to hospital presentation.^[11,12,21,22]^

#### 3.6.1. Primary outcome

##### 3.6.1.1. Survivability

Hossein et al demonstrated recovery in 50% of cases in the group receiving insulin, while only 27.3% could survive in the comparison group. Also, overall hospital stay in the former group was longer than in another group (median: 60 hours vs 24 hours).^[21]^ Similarly, Pannu et al showed a reduction in hospital mortality in the insulin-based treatment group (n = 14; 46.7%) compared to the control group (n = 22; 73.3 %). The median cumulative survival time was also longer, that is, 120 hours (CI, 0–240) as compared to 11 hours (CI, 5.8–16.8) [*P*-value .01].^[11]^ RCT study of Adel et al found a significant increase in survival, 35.2% versus 3.7%, and duration of hospital stay, 13 hours versus 7 hours, respectively, in the insulin group versus control group.^[22]^ The overall mortality rate was 61.4% in Nasrin et al’s study.^[12]^

Furthermore, regression analyses were performed to show the dosage effects of the insulin regimen on mortality. Hossein et al’s regression model (with a model significance of 0.032, Nagelkerke *R*^2^: 0.610) showed that the risk of mortality decreased by 4.5% with each hour of insulin infusion.^[21]^ Nasrin et al’s design of a logistic regression model (with an accuracy of 80%) between patients’ outcome and the last dose of insulin received showed an increase in the chance of patient’s survival by 1.028 (odds ratio; CI, 1.016–1.041) per 1 IU increase in insulin dose.^[12]^

#### 3.6.2. Secondary outcomes

Effect of insulin therapy on BP, blood pH, blood gasses, and need for mechanical ventilation. The authors had claimed the average values of hemodynamic parameters (SBP, diastolic blood pressure (DBP), MAP, blood pH, partial pressure of carbon dioxide [pCO2], and bicarbonate levels) were comparable between the 2 groups at the time of admission.^[11,21,22]^ Analysis at the end of the study showed variable results among studies, as mentioned in the following subtopics.

##### 3.6.2.1. Effect on BP

Where Hossein et al did not find any significant difference in DBP and statistically significant lower SBP in the insulin-based study group,^[21]^ other studies were consistent with the positive effect of insulin on BP. Pannu et al found a higher SBP, DBP, and MAP at 12, 24, and 48 hours after initiation of the insulin-based regimen compared to the control group with *P* < .001 for each component.^[11]^ Adel et al found that the insulin-based treatment is effective in increasing the SBP, DBP, and MAP at 6- and 12-hours post-admission compared to the placebo group and also demonstrated a lower requirement of vasopressor in the insulin-based group than the control group (with median dose: 7 mg vs 26 mg; *P* = .006).^[22]^ Nasrin et al showed a statistically significant role of insulin in raising the SBP by 0.02 mm Hg/IU insulin (*P* = .002; standard error: 0.1).^[12]^

##### 3.6.2.2. Correction of blood gasses

The authors also included parameters like blood pH, bicarbonate level, and pCO2 in arterial blood for comparison in their studies. Pannu et al and Hossein et al did not find any statistically significant role of insulin-based therapy in correcting the pH and blood gas derangement.^[11,21]^ Adel didn’t find any notable correction of blood pH and pCO2 with insulin use but demonstrated a significant increase in blood bicarbonate levels with insulin therapy at 6, 12, 18, and 24 hours (mean: 18 vs 13.8, 21.4 vs 16.2, 23.4 vs 18.3, 23.9 vs 14.2 mEq/L, respectively). They also demonstrated a linear decrease in mean serum lactate levels, which were 6.2 mmol/L at the time of admission. Traced 6 hourly as 3.5, 2.6, 2.2 to 1.7 at 24 hours and 1.3 mmol/L at 48 hours post admission.^[22]^ Nasrin et al found a corresponding decrease of 0.31 in the pH level for every 1 mIU increase in insulin.^[12]^

##### 3.6.2.3. Mechanical ventilation support

Hossein et al found no significant difference in the need for mechanical ventilation between the 2 groups. Forty-two participants in the control group required intubation and ventilation support and 39 in the insulin-based group.^[21]^ Pannu et al had reported a significant disparity in the need for mechanical ventilation between groups: 40.9% in the insulin therapy group compared to 60% in the control group (*P*-value < .01).^[11]^ Adel showed that 61.1% of patients receiving insulin required mechanical ventilation out of 54 symptomatic patients, whereas 81.5% of patients in the control group needed ventilation, indicating a significant difference.^[22]^ Nasrin et al did not conduct an analysis related to mechanical ventilation. Although the frequency of mechanical ventilation was lesser in the insulin based groups, neither of the studies mentioned the indications of intubation or whether the insulin regimen had delayed the need for mechanical ventilation.

##### 3.6.2.4. Insulin regimen-related complications

Two studies documented the effect on blood glucose and potassium levels in the study groups. Both authors claim measurement of blood sugar every hour and titrating dextrose infusion (dosage not mentioned) and serum potassium every 4 to 8 hours.^[11,22]^ Pannu et al found the incidence of hyperglycemia (> 200 mg/dL) was 53.3% among patients in the insulin group compared to 3.3% among the controls (*P*-value < .01). Hypoglycemia was also more common in the intervention group, but there was no statistically significant difference (20% versus 10%, *P*-value .47) compared to the control group.^[11]^ Adel et al found hypoglycemia in 2 among 54 participants in insulin-based treatment and in 9 of 54 of the control group; similarly, hypokalemia in 3 and 4 patients of respective groups.^[22]^

## 4. Discussion

The common hemodynamic features of AlP toxidrome are hypotension, decreased cardiac output, and bradycardia or tachycardia. The authors believe the pathophysiologic effects of phosphine toxicity could be the result of combined energy insufficiency as well as oxidative stress.^[23]^ The exact mechanism of action of insulin-based therapy on phosphine-toxin-induced myocardial injury is yet to be completely defined, but some of the already discovered ones are positive inotropy, improving myocyte carbohydrate uptake and utilization, and restoration of calcium flux.^[12,21]^ Insulin has also been shown to have antioxidant and protective properties in injured cardiomyocytes of animal and human models.^[24]^

Studies have shown a consistent result of decreased mortality in patients who have received insulin therapy. Adding exogenous insulin as a part of treatment has decreased mortality by 22.7% to 31.5%.^[11,21,22]^ However, there was a vast difference in the total dose of insulin required to achieve these results. The vast disparity in average insulin dosage among studies might be partially explained by the choice difference in practice of insulin use, that is, where Pannu et al and Adel et al used insulin in a fixed dosage regimen, Hossein et al used insulin also to titrate SBP > 90 and to correct metabolic acidosis.^[21]^

The studies reviewed in our paper showed variable results regarding the effect on BP, as mentioned above. The effects like increased myocardial contractility and sodium water retention, might increase BP. However, insulin has a dual effect on vascular tone. The vasoactive effect of insulin depends on the balance between vasodilation and vasoconstriction mediated by endothelial-derived nitric oxide and endothelin 1.^[21]^ Factors like oxidative stress, high plasma renin activity, low pH, and hyperphosphatemia in AlP poisoning may also interfere with the vasoactive action of insulin.^[25–29]^ A retrospective study by Dorooshi et al (n = 31) who used insulin in AlP poisoning cases with vasopressor-resistant hypotension found no significant relationship between the outcome and the total amount of insulin received, which might question the role of insulin in the most critical phase of toxidrome.^[28]^ Insulin therapy has not been shown to be much effective in correcting blood pH and arterial blood gas disorders.^[11,12,21]^ The disbalance in acid–base and blood gas physiology in AlP poisoning is probably due to a combination of tissue hypoperfusion and induced error in cellular metabolism. The rise in serum lactate in the first few hours of exposure to phosphine might also point to derangement in cellular metabolism.^[30,31]^ Among 4 studies, only B. Adel et al studied and showed correction of lactic acidosis in the insulin therapy group, which might be due to some correction of cellular glucose metabolism by the high dose of insulin. Studies have demonstrated the need for mechanical ventilation as a negative predictable factor of survivability in AlP poisoning.^[32,33]^ Also, the low need for ventilator support in any poisoning is another welfare for rural regions or the developing world where pesticide poisoning is a major concern and ventilators and intensive care unit facilities are not readily available.^[11]^ The studies have shown variable results: significant decrease^[11,22]^ to nonsignificant effect^[21]^ on the frequency of mechanical ventilation in groups with insulin-based therapy compared to conventional therapy groups.

Since 16 years after its initial appearance in medical literature,^[16]^ only a few interventional studies using insulin in AlP poisoning have been performed. Despite the favorable outcomes observed in most of these studies, we still lack the high-quality RCTs necessary for conducting meta-analyses and presenting a robust conclusion. If found beneficial, it would be a feasible, easy to implement, and economical therapy without much concerning adverse effects. Results of studies showing significant enhancements in survivability in AlP poisoning like the initial lavage with mineral oils,^[34]^ use of N-acetylcysteine,^[35]^ magnesium sulfate,^[36]^ lipid emulsion,^[37,38]^ ECMO (extracorporeal membrane oxygenation),^[39]^ etc, are admirable. The exploration of combination therapy involving these therapies and the search for modalities to increase the clearance of phosphine and its metabolites from the body is the subject of interest for future research. Although this article concludes without a definitive statement, it offers significant results. We, the authors, believe that this paper will inspire readers, future researchers, and authorities to contribute to this promising approach for combating AlP-related fatalities.

## 5. Conclusion

Insulin in the treatment of symptomatic AlP poisoning cases has been advocated as a promising entity multiple times in the past. Our review revealed a potential positive impact on survival with the use of exogenous insulin in these cases. This trend was consistently observed across all reviewed studies, yet the absence of some high-quality randomized control trials prevents us from asserting its efficacy with certainty. Therefore, confidently recommending its inclusion in therapy awaits further investigation and remains a noteworthy subject for future research.

## Acknowledgments

The authors would like to express immense gratitude to Professor Dr Vinutha Silvanus, Head of Department, Department of Community Medicine, Nepal Medical College and Teaching Hospital, Kathmandu, Nepal (ORCiD: 0000-0002-9559-001X) for her valuable suggestions and thorough guidance.

## Author contributions

**Conceptualization:** Ravi Shukla.

**Data curation:** Ravi Shukla, Kiran Lamichhane, Dhritee Pandey.

**Formal analysis:** Dhritee Pandey, Sashank Shukla.

**Methodology:** Ravi Shukla, Kiran Lamichhane.

**Resources:** Chandan Kumar Gupta.

**Software:** Kiran Lamichhane, Chandan Kumar Gupta.

**Supervision:** Dhritee Pandey, Sashank Shukla.

**Validation:** Dhritee Pandey, Sashank Shukla.

**Visualization:** Ravi Shukla.

**Writing** – **original draft:** Ravi Shukla.

**Writing** – **review & editing:** Ravi Shukla, Kiran Lamichhane, Dhritee Pandey, Chandan Kumar Gupta.

## Supplementary Material


