# Supplementary material for: Insulin in aluminum phosphide poisoning: A systematic review of the current literature
Source: Medicine (Baltimore). 2024 Oct 18;103(42):e40066. doi: 10.1097/MD.0000000000040066 (PMC11495704; doi:10.1097/MD.0000000000040066)
Supplement: Supplementary file 1 [file medi-103-e40066-s001.docx]

# Appendix

# Search strategy:

### PubMed Central

#1 “Insulin”[MESH]

#2 “Insulin-euglycemia therapy” OR “GIK solution” OR “glucose insulin potassium” OR “hyperinsulinemia euglycemia therapy” OR HIET OR HDI OR “high dose insulin”

#3 “aluminum phosphide"[All Fields] OR aluminum phosphide[Text Word] OR phosphines OR AIP OR AP OR Fumitoxin OR Phostek OR Delicia OR Fosfuri di alluminio OR rice tablet OR Alphos OR Celphos OR Quick phos OR Phostoxin OR Delicia gastoxin

Final search: (#1 OR #2) AND #3

Search filter: Humans

Fields: All fields

Date of search: 15/02/2024

Number of results: 745 results

### Cochrane Library

#1 Insulin OR Insulin-euglycemia therapy OR GIK solution OR glucose insulin potassium OR hyperinsulinemia euglycemia therapy OR hyperinsulinemia euglycemic therapy OR HIET OR HDI OR high dose insulin

#2 Aluminium Phosphide OR phosphines OR “aluminum phosphide” OR AIP OR AP OR alumanylidynephosphane OR Fumitoxin OR Phostek OR Celphine OR Celphide OR “Aluminium fosfide” OR “Detia gas Ex-B” OR Delicia OR Fosfuri di alluminio OR rice tablet OR Alphos OR Celphos OR Quick phos OR Phostoxin OR Delicia gastoxin

Search filter options: “All text” for #1 AND “Title Abstract keyword” for #2

Fields: All fields

Limitations: no limitations applied

Date of search: 15/02/2024

Number of results: 328 results

### Google scholar

insulin "rice tablet" OR "Aluminum phosphide" OR "Aluminum phosphide" OR Alphos OR Celphos OR Celphide OR alumanylidynephosphane OR Fumitoxin OR Phostek OR Celphine OR Celphide OR "Aluminum fosfide" OR "Detia gas Ex B" OR Delicia OR "Fosfuri di alluminio" OR "Quick phos" OR Phostoxin OR "Delicia gastoxin"

Fields: All fields

Limitations: None

Date of search: 15/02/2024

Number of results: 925 results

## Quality appraisal tools:

Table I: ROBINS-I tool (Risk Of Bias In Non-randomized Studies - of Interventions) for quasi-randomized studies of Hossein et al. and Pannu et al.

| Domains of bias (ROBINS-I) | **Hossein et al.** | **Pannu et al.** |
| --- | --- | --- |
| Bias due to confounding: | “Low risk” | “Low risk” |
| Bias in selection of participants into the study | “Low risk” | “Low risk” |
| Bias in classification of interventions: | “Moderate risk” | “Low risk” |
| Bias due to deviations from intended interventions | “Moderate risk” | “Moderate risk” |
| Bias due to missing data | “Low risk” | “Low risk” |
| Bias in measurement of outcomes | “Serious risk” | “Serious risk” |
| Bias in selection of the reported result: | “Low risk” | “Low risk” |
| Overall bias | “Serious risk” | “Serious risk” |

Table II: The Revised Cochrane risk-of-bias tool for randomized trials (RoB 2) used for the RCT study of Adel et al.

| S.No. | Domains of bias (Rob 2) | Comment |
| --- | --- | --- |
| 1. | Risk of bias arising from the randomization process: | “Low risk” |
| 2. | Risk of bias due to deviations from the intended interventions : | “Some concerns” |
| 3. | Risk of bias due to missing outcome data: | “Low risk” |
| 4. | Risk of bias in measurement of the outcome: | “Some concerns” |
| 5. | Risk of bias in selection of the reported result: | “Low risk” |
|  | Overall risk of bias: | “Some concerns” |

Table III: NHLBI Quality Assessment Tool for Before-After (Pre-Post) Studies With No Control Group was used for the longitudinal interventional study of Nasrin et al.

| S No. | Quality Assessment Tool for Before-After (Pre-Post) Studies With No Control Group | Comment |
| --- | --- | --- |
| 1. | Was the study question or objective clearly stated? | Yes |
| 2. | Were eligibility/selection criteria for the study population prespecified and clearly described? | Yes |
| 3. | Were the participants in the study representative of those who would be eligible for the test/service/intervention in the general or clinical population of interest? | Yes |
| 4. | Were all eligible participants that met the prespecified entry criteria enrolled? | Yes |
| 5. | Was the sample size sufficiently large to provide confidence in the findings? | Yes |
| 6. | Was the test/service/intervention clearly described and delivered consistently across the study population? | Yes |
| 7. | Were the outcome measures prespecified, clearly defined, valid, reliable, and assessed consistently across all study participants? | Yes |
| 8. | Were the people assessing the outcomes blinded to the participants' exposures/interventions? | Yes |
| 9. | Was the loss to follow-up after baseline 20% or less? Were those lost to follow-up accounted for in the analysis? | Yes |
| 10. | Did the statistical methods examine changes in outcome measures from before to after the intervention? Were statistical tests done that provided p values for the pre-to-post changes? | Yes |
| 11. | Were outcome measures of interest taken multiple times before the intervention and multiple times after the intervention (i.e., did they use an interrupted time-series design)? | Yes |
| 12. | If the intervention was conducted at a group level (e.g., a whole hospital, a community, etc.) did the statistical analysis take into account the use of individual-level data to determine effects at the group level? | Yes |
| 13. | Quality Rating | Good |

Rater #1: Ravi Shukla

Rater #2: Kiran Lamichhane

Arbiter: Dhritee Pandey
